# Oesophageal cancer awareness and anticipated time to help-seeking: results from a population-based survey

**DOI:** 10.1038/s41416-024-02663-1

**Published:** 2024-03-30

**Authors:** Jasmijn Sijben, Lotte J. Huibertse, Linda Rainey, Mireille J. M. Broeders, Yonne Peters, Peter D. Siersema

**Affiliations:** 1 Department of Gastroenterology and Hepatology, Radboud university medical center, Nijmegen, The Netherlands; 2 IQ Health Science Department, Radboud university medical center, Nijmegen, The Netherlands; 3 Dutch Expert Centre for Screening, Nijmegen, The Netherlands; 4 Department of Gastroenterology and Hepatology, Erasmus MC - University Medical Center, Rotterdam, The Netherlands

**Keywords:** Digestive signs and symptoms, Oesophageal cancer, Patient education

## Abstract

**Background:**

Modifying public awareness of oesophageal cancer symptoms might help to decrease late-stage diagnosis and, in turn, improve cancer outcomes. This study aimed to explore oesophageal cancer symptom awareness and determinants of lower awareness and anticipated time to help-seeking.

**Methods:**

We invited 18,156 individuals aged 18 to 75 years using random sampling of the nationwide Dutch population registry. A cross-sectional web-based survey containing items adapted from the Awareness and Beliefs about Cancer measure (i.e., cancer symptom awareness, anticipated time to presentation with dysphagia, health beliefs, and sociodemographic variables) was filled out by 3106 participants (response rate: 17%). Descriptive statistics were calculated and logistic regression analyses were performed to explore determinants of awareness and anticipated presentation (dichotomised as <1 month or ≥1 month).

**Results:**

The number of participants that recognised dysphagia as a potential symptom of cancer was low (47%) compared with symptoms of other cancer types (change in bowel habits: 77%; change of a mole: 93%; breast lump: 93%). In multivariable analyses, non-recognition of dysphagia was associated with male gender (OR 0.50, 95% CI 0.43−0.58), lower education (OR 0.44, 0.35−0.54), and non-western migration background (OR 0.43, 0.28−0.67). Anticipated delayed help-seeking for dysphagia was associated with not recognising it as possible cancer symptom (OR 1.58, 1.27−1.97), perceived high risk of oesophageal cancer (OR 2.20, 1.39−3.47), and negative beliefs about oesophageal cancer (OR 1.86, 1.20−2.87).

**Conclusion:**

Our findings demonstrate a disconcertingly low public awareness of oesophageal cancer symptoms. Educational interventions targeting groups with decreased awareness and addressing negative cancer beliefs may lead to faster help-seeking behaviour, although additional studies are needed to determine the effect on clinical cancer outcomes.

## Introduction

The incidence of oesophageal cancer is expected to rise dramatically across European and North American counties in the coming years [1]. The prognosis of oesophageal cancer is poor and mainly related to the stage at diagnosis [2, 3]. When diagnosed at stage I, the three-year survival rate of oesophageal cancer is 46%−83%, while if diagnosed at stage IV, it is as low as 3%−5% [4]. Unfortunately, approximately 40% of patients with oesophageal cancer are diagnosed at stage IV [4, 5]. Minimising delays across the diagnostic pathway might reduce late-stage diagnosis [6].

The Model of Pathways to Treatment describes that patient, healthcare system, and disease factors interact to affect diagnostic interval lengths [7]. Previous studies found that the patient interval (the time between first noticing a symptom to consultation in primary care) is most prolonged in the diagnostic pathway of oesophageal cancer [8–11]. Studies in patients with oesophageal cancer have indicated that non-recognition of dysphagia as a suspicious symptom was the predominant factor in delayed symptomatic presentation [12–14]. Some patients normalised or misattributed dysphagia as a usual bodily reaction, thinking they ‘swallowed food the wrong way’ or ‘did not chew the food properly’ and thus did not perceive a reason to seek medical help [12–14]. Educational interventions may stimulate earlier help-seeking behaviour once symptoms arise, aiming to decrease late-stage diagnosis and, in turn, improve cancer outcomes.

Insight in public awareness of oesophageal cancer symptoms and help-seeking intentions is critical to develop interventions aimed at reducing the patient interval. Prior surveys of the general public of Ireland and the United Kingdom (UK) showed a poor level of awareness that dysphagia may be a sign of cancer [15, 16], in correspondence with the studies conducted among patients. However, the current literature does not provide information on sociodemographic and psychological indicators of oesophageal cancer awareness and anticipated help-seeking. This information could help suggest how to target those most at risk of delaying care and which messages to incorporate. In addition, insight in sociodemographic patterns of help-seeking could shed a light on the hypothesis that patient delays contribute to the poorer outcomes observed among oesophageal cancer patients with lower socio-economic status (SES) [17].

We aimed to identify levels of oesophageal cancer symptom awareness and sociodemographic determinants of lower awareness and anticipated delay in the general Dutch population. We also examined the effects of health beliefs on anticipated delay, including perceived personal risk and the presence of negative beliefs about oesophageal cancer [18].

## Methods

### Design and setting

Cross-sectional data were collected in the Netherlands between January and September 2023 using a web-based survey. The present survey was administered simultaneously with another survey estimating the prevalence of gastro-oesophageal reflux disease (GORD) symptoms. The study was conducted among the Dutch general population. Ethics approval was acquired from the regional ethics committee METC Oost-Nederland in the Netherlands (Ref no. 2022-13720). Informed consent was obtained online prior to the start of the survey. The protocol for this study has been registered in ClinicalTrials.gov (NCT05689918). The survey was conducted and is reported in accordance with the checklist for Reporting Results of Internet E-Surveys (CHERRIES) [19].

### Participants

**Fig. 1:**
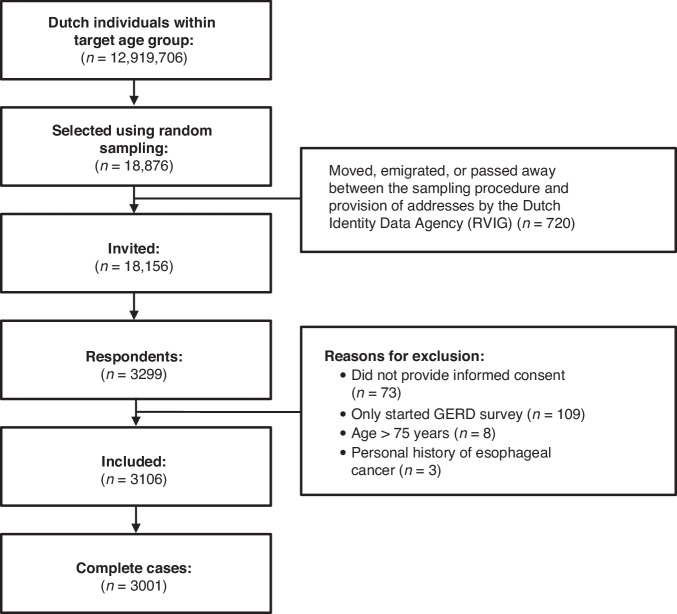
Study flow. Flow chart of study participants. GORD gastro-oesophageal reflux disease.

Participants were eligible if they were between 18 and 75 years of age and had access to the survey using a phone, tablet, or computer. Simple random sampling was performed in the Dutch population registry to select individuals in the target age group (Fig. 1). Selected individuals were sent an invitation letter through postal mail containing a URL and corresponding QR-code to the survey, which was linked to a unique participant identification number to prevent duplicate entries. We did not prevent participants with a history of oesophageal cancer from completing the survey, but their data were subsequently excluded from analyses.

National demographics acquired from the Dutch Central bureau of Statistics (CBS) (age, sex, education, and migration background) were compared with the distribution of these variables in the study sample (Table 1).

**Table 1:** Demographic characteristics of participants compared with Dutch population statistics.

	Participants (*N* = 3106)		Dutch population (*N* = 12,919,706)^f^
	*n*	%	%^f^
Age (years), *n* (%)^a^			
18−24	207	6.7	12.1
25−34	317	10.2	17.7
35−44	372	12.0	16.4
45−54	537	17.3	18.3
55−64	767	24.7	18.8
65−75	832	26.8	16.7
Gender, *n* (%)^a^			
Male	1523	49.0	50.1
Female	1502	48.4	49.9
Non-binary	7	0.2	NA
Education level, *n* (%)^b^			
Lower	534	17.2	22.6^g^
Middle	1060	34.1	35.7^g^
Higher	1436	46.2	40.9^g^
Civil status, n with a partner (%)^b^	2271	73.1	NA
SES score neighbourhood, *n* (%)^c,h^			
< −0.2 (most deprived)	312	10.1	NA
−0.2 to −0.1	236	7.6	NA
−0.1 to 0	335	10.8	NA
0 to 0.1	477	15.4	NA
0.1 to 0.2	584	18.8	NA
>0.2 (least deprived)	752	24.2	NA
Migration background, *n* (%)^b^			
Dutch background	2856	92.0	74.1
Western migration background	73	2.4	11.5
Non-western migration background	101	3.2	14.4
Knew someone with oesophageal cancer, *n* yes (%)^d^	456	14.7	NA
Perceived risk of oesophageal cancer, *n* (%)^e^			
Very low	515	16.6	NA
Somewhat low	1142	36.8	NA
Moderate	1278	41.1	NA
Somewhat high	100	3.2	NA
Very high	10	0.3	NA

*SES* socio-economic status, *NA* not available.

^a^*n* = 74 missing values (2.4%).

^b^*n* = 76 missing values (2.4%).

^c^*n* = 409 missing values (13.2%).

^d^*n* = 102 missing values (3.3%).

^e^*n* = 61 missing values (2.0%).

^f^of population aged 18−75 years on 1 January 2022.

^g^% of population aged 25−74 years on 1 January 2022 (*n* = 11,038 *1000).

^h^Score for the average socio-economic status in the participant’s neighbourhood based on financial welfare, education and employment, calculated by statistics Netherlands.

### Survey instrument

Survey items were adapted from the generic Awareness and Beliefs about Cancer (ABC) Measure, which is validated among the general populations of six countries (the UK, Australia, Canada, Sweden, Denmark, and Norway) [20]. Items were made specific for oesophageal cancer (described below) and reviewed by professionals (a gastroenterologist, epidemiologist, psychologist, general practitioner, and representatives from the patient support group). The survey was subsequently piloted in cognitive interviews (*n* = 7) and tested for technical functionality in the Castor Electronic Data Capture (EDC) platform (*n* = 3) [21]. The total survey took 10−15 min to complete (including the GORD prevalence survey). Participants directly entered data in Castor EDC. An English translation of the Dutch survey is available in the supplementary material.

### Awareness of cancer symptoms

Unprompted awareness of oesophageal cancer symptoms was first assessed with an open-ended item adapted from the ABC questionnaire [20]: ‘There are many signs and symptoms of oesophageal cancer. Please name as many as you can think of’. Closed recognition items from the ABC-questionnaire using the item ‘Do you think X could be a sign of cancer?’ were then administered to measure prompted awareness of cancer symptoms. The term ‘dysphagia’ used throughout this article entails both the symptoms ‘difficulty swallowing’ and ‘food obstruction’.

### Anticipated time to help-seeking

Anticipated time to help-seeking for dysphagia was assessed by adapting one item from the ABC questionnaire: ‘If you persistently had the sensation of food getting caught after you’ve started to swallow, how long would it take you to go to the doctor from the time you first noticed the symptom?’. Responses were recoded into early anticipated help-seeking (I would go as soon as I noticed, up to 1 week, over 1 up to 2 weeks, over 2 up to 3 weeks, over 3 up to 4 weeks), delayed anticipated help-seeking (more than a month, I would not contact my doctor) or missing (I would go to a pharmacist). No standardised guidelines are available about when to seek help for dysphagia. The cut-off of 1 month was chosen because it exceeds the median patient interval of 29 days among oesophageal cancer patients [8].

### Beliefs about oesophageal cancer

The beliefs about cancer module in the ABC questionnaire was adapted to assess six beliefs about oesophageal cancer outcomes and the value of early presentation [20]. Answers were provided on a 4-point Likert scale ranging from 1 (strongly disagree) to 4 (strongly agree). Table 2 presents the questions and how these were classified. Perceived oesophageal cancer risk was assessed using the item ‘How do you perceive your own risk of developing oesophageal cancer in comparison with someone of your age?’, with five response options (low, below average, average, above average, and high).

**Table 2:** Questions about oesophageal cancer beliefs, responses, and how these were classified.

	Strongly agree or tend to agree, *n* (%)	Strongly disagree or tend to disagree, *n* (%)
‘These days, many people with oesophageal cancer can expect to continue with normal activities and responsibilities’^a^	1571 (50.6)	**1487 (47.9)**
‘Oesophageal cancer can often be cured’^b^	1360 (43.8)	**1693 (54.5)**
‘Going to the doctor as quickly as possible after noticing a symptom of oesophageal cancer could increase the chances of surviving’^c^	2874 (92.5)	**175 (5.6)**
‘Oesophageal cancer treatment is worse than the cancer itself’^b^	**1042 (33.5)**	2011 (64.7)
‘I would NOT want to know if I have oesophageal cancer’^d^	**290 (9.3)**	2757 (88.8)
‘Oesophageal cancer is a death sentence’^d^	**888 (28.6)**	2159 (69.5)

Answers classified as negative oesophageal cancer beliefs are highlighted in bold.

^a^*n* = 48 missing values (1.5%).

^b^*n* = 53 missing values (1.7%).

^c^*n* = 57 missing values (1.8%).

^d^*n* = 59 missing values (1.9%).

### Sociodemographic background

Data on participants’ age, gender, education attainment (lower education, middle education, higher education), civil status (with vs. without a partner), migration background (not migrated, western migration background, non-western migration background), and knowing someone affected by oesophageal cancer (yes vs. no or do not know) were acquired. We also used participants’ self-administered postal codes to obtain the average SES score of their neighbourhood as calculated by the CBS. This score ranges from -0.9 to 0.9, with 0 being the Dutch average, and is based on financial welfare, education attainment, and employment [22].

### Sample size

Sample size calculations were based on prior estimates of the primary outcome of each survey (i.e., the present survey and the survey estimating GORD symptom prevalence that was sent simultaneously) and the largest required sample size was selected. A sample size of 4719 was found to be sufficient to estimate the prevalence of GORD symptoms with a confidence level of 99% and 1.5% margin of error. A total of 18,876 individuals were sampled based on an assumed participation rate of 25%. The actual participation rate of 17% yielded a final sample of 3106 participants, which is sufficient to estimate the proportion of Dutch individuals recognising oesophageal cancer symptoms with a confidence level of 95% and 2% margin of error.

### Data analyses

Descriptive statistics were calculated and reported as *n* (%). Responses to the open-ended item were analysed using the list of ten oesophageal cancer symptoms described on the Dutch lay medical education platform created by the Dutch Association of General Practitioners [23], by recoding the response into a binary outcome for each of the ten symptoms (did mention vs. did not mention). Univariable and multivariable logistic regression analyses were performed to identify determinants of recognition of dysphagia as cancer symptom and anticipated time to help-seeking for dysphagia. Recognition of dysphagia was treated as an independent variable in the model of anticipated help-seeking. Determinants with a *p* value of < 0.2 in univariable analyses were included in the multivariable model, followed by backward elimination of non-significant variables (stopping rule: *p* < 0.05). Because of the relatively small proportion of missing data (not exceeding 3.3% except for the non-mandatory item postal code), their impact on the estimates is likely to be marginal. We used a complete case analysis approach for logistic regression analyses and reported the number of missing values for descriptive statistics. Data were analysed using SPSS version 27. Significance tests were two-sided; *p* < 0.05 was considered statistically significant.

## Results

### Characteristics and representativeness of the study sample

Figure 1 shows that of the 18,876 sampled individuals, 720 moved, emigrated, or passed away before addresses were provided, leaving a total of 18,156 invitations sent. A total of 3106 eligible participants returned the survey (response rate: 17%). Participants were excluded if they did not provide informed consent (*n* = 73), only started the survey about GORD symptoms that was sent simultaneously (*n* = 109), were not in the target age group (*n* = 8) or had a history of oesophageal cancer (*n* = 3). Gender distribution did not differ between the sample and Dutch population statistics, but persons who were older, had a higher educational level, higher SES score, and were born in the Netherlands were overrepresented in the study sample (Table 1). Moreover, 14.7% reported knowing someone with oesophageal cancer.

### Awareness of cancer symptoms

**Fig. 2:**
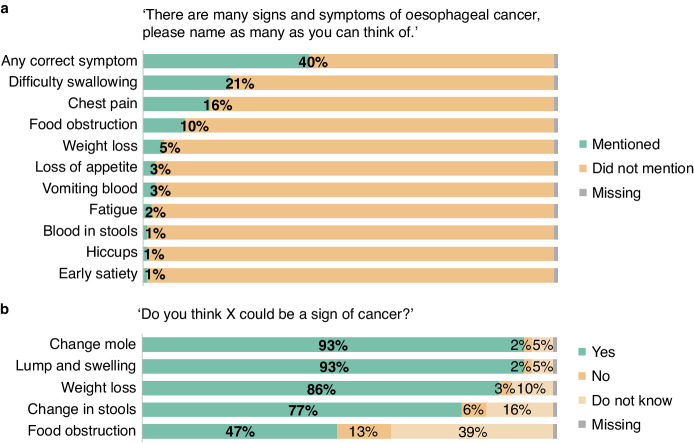
Awareness of cancer symptoms. **a** Response to open question eliciting unprompted awareness of oesophageal cancer symptoms. **b** Response to recognition item eliciting prompted awareness of oesophageal cancer-specific, generic, and comparator cancer symptoms.

Figure 2a shows responses to the open (unprompted) item investigating oesophageal cancer symptom awareness. Notably, 59.7% did not mention any correct symptom. Participants most frequently mentioned ‘difficulty swallowing’ (21.3%), ‘chest pain’ (16.4%), and ‘food obstruction’ (10.6%), and least frequently mentioned ‘early satiety’ (0.5%) as potential symptoms of oesophageal cancer.

The proportion that recognised ‘food obstruction’ as a potential cancer symptom was higher with the closed (prompted) item (47.2%) (Fig. 2b). However, awareness of symptoms associated with other cancer types was markedly higher: ‘a change in the appearance of a mole’ for melanoma, ‘an unexplained lump or swelling’ for breast cancer, and ‘a change in bowel or bladder habits’ for colorectal and bladder cancer, were recognised by 93.0%, 92.6%, and 77.0% of the participants, respectively.

In multivariable analysis, lower education (OR 0.44, 95% CI 0.35−0.54), male gender (OR 0.50, 95% CI 0.43−0.58), non-western migration background (OR 0.43, 95% CI 0.28−0.67), and not knowing someone with oesophageal cancer (OR 0.53, 95% CI 0.43−0.66) were associated with a decreased likelihood to recognise dysphagia as a potential cancer symptom (Table 3).

**Table 3:** Explorative analysis of determinants associated with recognition of dysphagia as potential cancer symptom.

	Recognised symptom (%)	Univariable OR (95% CI)	*P* value	Multivariable^a^ OR (95% CI)	*P* value
Age		0.99 (0.99−0.99)	0.008		
Gender					
Female	55.9	ref		ref	
Male	39.8	0.52 (0.45−0.60)	<0.001	0.50 (0.43−0.58)	<0.001
Education level					
Lower	35.0	0.47 (0.38−0.57)		0.44 (0.35−0.54)	<0.001
Middle	46.3	0.75 (0.64−0.88)	<0.001	0.74 (0.63−0.87)	<0.001
Higher	53.6	ref	<0.001	ref	
Civil status					
With a partner	48.0	ref			
Without a partner	47.3	0.97 (0.83−1.15)	0.76		
SES score neighbourhood		1.55 (1.09−2.20)	0.02		
Migration background					
Dutch background	48.6	ref		ref	
Western migration background	42.5	0.78 (0.49−1.25)	0.30	0.69 (0.43−1.13)	0.14
Non-Western migration background	29.7	0.45 (0.29−0.69)	<0.001	0.43 (0.28−0.67)	<0.001
Knew someone with oesophageal cancer					
Yes	61.4	ref		ref	
No/do not know	45.6	0.53 (0.43−0.65)	<0.001	0.53 (0.43−0.66)	<0.001

*SES* socio-economic status.

^a^Determinants with a *p* value of < 0.2 in univariable analyses were included in the multivariable model, followed by backward elimination of non-significant variables (stopping rule: *p* < 0.05).

### Anticipated time to help-seeking

**Fig. 3:**
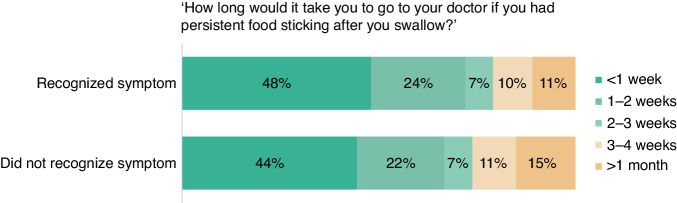
Anticipated time to help-seeking. Anticipated time to help-seeking for dysphagia, stratified by recognition that this may be a symptom of cancer.

Figure 3 shows participants’ anticipated time to help-seeking for dysphagia. Increased likelihood of delayed anticipated help-seeking for dysphagia was associated with not recognising it as a possible symptom of cancer (OR 1.58, 95% CI 1.27−1.97), perceived high risk of oesophageal cancer (OR 2.20, 95% CI 1.39−3.47), and expressing ≥ 4 negative beliefs about oesophageal cancer (OR 1.86, 95% CI 1.20−2.87) (Table 4).

**Table 4:** Explorative analysis of determinants associated with delayed anticipated help-seeking for dysphagia ( ≥ 1 month).

	Delayed anticipated help-seeking (%)	Univariable OR (95% CI)	*P* value	Multivariable OR (95% CI)	*P* value
Age		0.98 (0.97−0.99)	<0.001	0.98 (0.98−0.99)	<0.001
Gender					
Male	11.8	ref			
Female	14.2	1.23 (1.00−1.53)	0.05		
Education					
Lower	12.7	ref			
Middle	11.8	0.92 (0.67−1.26)	0.59		
Higher	13.9	1.11 (0.83−1.49)	0.49		
Civil status					
With a partner	12.5	ref			
Without a partner	14.5	1.19 (0.94−1.51)	0.15		
SES score neighbourhood		0.82 (0.49−1.37)	0.45		
Migration background					
Dutch background	13.0	ref		ref	
Western migration background	17.8	1.45 (0.79−2.66)	0.24	1.30 (0.70−2.42)	0.40
Non-Western migration background	7.9	0.57 (0.28−1.19)	0.14	0.45 (0.21−0.93)	0.03
Knew someone with oesophageal cancer					
Yes	14.3	ref			
No/do not know	12.8	0.88 (0.66−1.18)	0.39		
Recognition of dysphagia as cancer symptom					
Yes	10.8	ref		ref	
No/do not know	15.3	1.49 (1.20−1.85)	<0.001	1.58 (1.27−1.97)	<0.001
Perceived risk of oesophageal cancer					
Very low/somewhat low/ moderate	12.5	ref		ref	
Somewhat high/very high	24.5	2.28 (1.46−3.56)	<0.001	2.20 (1.39−3.47)	<0.001
Number of negative beliefs about oesophageal cancer					
0	11.3	ref		ref	
1	12.1	1.08 (0.76−1.54)	0.67	1.17 (0.82−1.68)	0.38
2	12.6	1.14 (0.80−1.61)	0.47	1.23 (0.86−1.75)	0.25
3	17.3	1.27 (0.88−1.83)	0.20	1.40 (0.96−2.02)	0.08
≥4	17.3	1.65 (1.07−2.52)	0.02	1.86 (1.20−2.87)	0.006

^a^Determinants with a *p* value of < 0.2 in univariable analyses were included in the multivariable model, followed by backward elimination of non-significant variables (stopping rule: *p* < 0.05).

## Discussion

This population-based survey found that symptoms of oesophageal cancer are not well-recognised by the general population. Men, people with lower education, and a non-western migration background were less likely to recognise dysphagia as potential cancer symptom. Anticipated delayed help-seeking for dysphagia was modestly associated with symptom non-recognition.

### Comparison with previous findings

Our findings demonstrate, consistent with prior literature, that recognition of dysphagia as a potential cancer symptom is substantially lower than recognition of ‘a change in the appearance of a mole’ or ‘an unexplained breast lump or swelling’ [15, 16]. This may reflect that national campaigns have focused on skin and breast cancers, suggesting that campaigns addressing oesophageal cancer symptoms may help the population appraise these more accurately as well. The potential of awareness campaigns is supported by a recent survey conducted in the UK that reported a much higher level of oesophageal cancer symptom awareness than our study (78% vs. 47% recognised difficulty swallowing as a potential cancer symptom) [24], which could be an effect of the 2015 oesophago-gastric ‘Be Clear on Cancer’ campaign [25].

The modest association we found between symptom recognition and prompt intended help-seeking may reflect the ambiguity of oesophageal cancer symptoms. Previous surveys also reported a stronger association for typically alarming symptoms such as breast changes or rectal bleeding, compared with more usual symptoms such as persistent cough or persistent pelvic pain [26, 27]. Dysphagia can be interpreted as a result of chewing or swallowing food the wrong way [14], which may lead people to be more inclined to adopt a wait-and-see approach even if they are aware that the symptom might be a sign of cancer.

The process of help-seeking is multifaceted and may, next to the appraisal of symptoms, also be influenced by sociodemographic and psychological factors [7]. Notably, SES and migration background were not associated with anticipated delay in our study. Our results thus do not support the hypothesis that socio-economic disparities in oesophageal cancer prognosis are related to longer patient intervals [17], although the hypothetical time to help-seeking we assessed might not mirror the barriers an individual might encounter in reality. We did identify an association between having negative beliefs about oesophageal cancer and delayed help-seeking intentions, which is consistent with previous surveys [27, 28]. Negative beliefs about the disease and its treatment may induce fatalistic cognitions (e.g., no action will be effective dealing with the disease), which will induce fear control processes such as denial and downplay of symptoms, leading to postponed help-seeking [29, 30]. Modifying negative beliefs about oesophageal cancer using educational interventions is particularly complicated since prognosis is in fact very poor. The emphasis should be placed on the significantly better outcomes associated with early detection as opposed to late detection.

### Implications

This study shows substantial room for improvement in public awareness of oesophageal cancer symptoms. However, prior literature raises the question of whether increased awareness will result in better cancer outcomes. The previously mentioned ‘Be Clear on Cancer’ campaign did not significantly improve cancer staging or 1-year survival [31]. In addition, while some studies found an association between shorter time to diagnosis and lower stage oesophageal cancer [6], other studies reported that time to diagnosis did not affect cancer stage, tumour resectability, postoperative morbidity or survival [6, 32]. These inconsistent findings might be explained by two types of unaddressed bias. First, it is challenging to ascertain the timepoint of symptom onset in retrospect since this information is not systematically registered and is prone to recall bias. Second, the available studies did not correct for the *waiting time paradox*, which suggests that disease factors confound the diagnostic interval (i.e., prompt investigation of seriously ill patients) [33]. However, it is also possible that early identifiable symptoms simply do not occur in enough patients. Dysphagia, the most common presenting symptom of oesophageal cancer, generally occurs only after significant intraluminal obstruction or infiltration of the myenteric plexus (late stage disease) [34].

Despite these inconclusive studies, we believe that the possibility of a favourable effect of awareness-raising strategies should not be ruled out. The key message of the ‘Be Clear on Cancer’ campaign was **‘**Having heartburn, most days, for 3 weeks or more could be a sign of cancer – tell your doctor’ [25]. Informing the public that progressive dysphagia could be a sign of cancer might be more effective since this symptom has the highest positive predictive value for oesophageal cancer [35]. It is also advisable to target groups most at risk of failing to recognise oesophageal cancer symptoms. Our study identified that these groups comprise men, lower-educated people, and those with non-western migration backgrounds, consistent with previous surveys [36–38]. Educational initiatives could target public understanding of male gender/risk association for oesophageal cancer and should have a multilingual and cultural design. Next to government-led campaigns, which may be costly, awareness-raising strategies might involve social media actions and advertising resources in general practice clinics. Since awareness interventions may also inflict undesirable side-effects, such as an unreasonable demand for gastroscopies [31], the challenge lies in guiding individuals through the symptom appraisal process. This could be done by introducing help-seeking decision-aids containing questions regarding the duration, progressiveness, and, particularly, the persistence of dysphagia. Furthermore, public interventions could be combined with symptom prediction models to assist general practitioners’ referral decisions [39].

### Strengths and limitations

A key strength of the present study was the use of the Dutch population registry, in which all Dutch residents are registered, enabling us to define a representative sample of the entire Dutch population aged 18 to 75 years. Furthermore, we incorporated previously validated questions in the survey whenever possible and tested the survey with the support of public and patient support group representatives. The study also has limitations that should be considered. Only 17% of the people we invited agreed to participate. Unfortunately, response rates of surveys have been declining over the past decades [40]. Generalisations obtained from our data should be made with caution as our sample characteristics differed from the Dutch population at large (Table 1). People with higher education, at older age, those from affluent areas, and those born in the Netherlands were overrepresented in our sample. Selective participation may have affected the findings since people born in the Netherlands, with a higher-level education, were also generally more aware of oesophageal cancer symptoms. Consequently, the actual awareness level in the population is likely even lower than estimated here. Selection bias related to the web-based design (i.e., individuals more comfortable with technology agreeing to participate) and language barriers (i.e., survey was only available in Dutch) may also have occurred. Furthermore, no validated measure of awareness and beliefs specific for oesophageal cancer was available. Therefore, we adapted items from the validated ABC questionnaire with minimal changes (e.g., ‘cancer’ was replaced by ‘oesophageal cancer’) and performed cognitive interviews to guarantee appropriate survey items.

## Conclusion

Our findings demonstrate a disconcertingly low public awareness of oesophageal cancer symptoms. Targeted awareness campaigns to improve recognition of dysphagia as a potential symptom of oesophageal cancer may improve earlier help-seeking. Additional studies are required to determine if awareness-raising strategies can affect clinical cancer outcomes without increasing inappropriate help-seeking.

### Supplementary information

Supplementary file

## Data Availability

Both datasets and scripts used to generate the analyses in this study are available at 10.17026/LS/LUXPJM.
