# Supplementary material for: Oesophageal cancer awareness and anticipated time to help-seeking: results from a population-based survey
Source: Br J Cancer. 2024 Mar 30;130(11):1795–802. doi: 10.1038/s41416-024-02663-1 (PMC11130305; doi:10.1038/s41416-024-02663-1)
Supplement: Supplementary file 1 — Supplementary file [file 41416_2024_2663_MOESM1_ESM.docx]

Survey part II

Signs of oesophageal cancer

We would like to ask a few questions about your awareness of cancer.

**1 There are many signs and symptoms of oesophageal cancer. Please name as many as you can think of:**

Signs of cancer

The following may or may not be signs of cancer. We are interested in your opinion, please do not use the internet:

**2. Do you think persistently having the sensation of food getting caught after you’ve started to swallow could be a sign of cancer?**

□ Yes
□ No
□ Don’t know

**3. Do you think a persistent change in bowel or bladder habits could be a sign of cancer?**

□ Yes
□ No
□ Don’t know

**4. Do you think a change in the appearance of a mole could be a sign of cancer?**

□ Yes
□ No
□ Don’t know

**5. Do you think unexplained weight loss could be a sign of cancer?**

□ Yes
□ No
□ Don’t know

**6. Do you think an unexplained lump or swelling could be a sign of cancer?**

□ Yes
□ No
□ Don’t know

**7. If you persistently had the sensation of food getting caught after you’ve started to swallow, how long would it take you to go to the doctor from the time you first noticed the symptom?**

□ I would go as soon as I noticed
□ Up to 1 week
□ Over 1 up to 2 weeks
□ Over 2 up to 3 weeks
□ Over 3 up to 4 weeks
□ More than a month
□ I would go to a pharmacist instead of a doctor
□ I would not contact my doctor

Risk factors

How much do you agree that each of these can increase a person’s chance of developing oesophageal cancer?

**8. How much do you agree that drinking alcohol can increase a person’s chance of developing oesophageal cancer?**

□ Strongly disagree
□ Tend to disagree
□ Tend to agree
□ Strongly agree

**9. How much do you agree that smoking can increase a person’s chance of developing oesophageal cancer?**

□ Strongly disagree
□ Tend to disagree
□ Tend to agree
□ Strongly agree

**10. How much do you agree that being overweight can increase a person’s chance of developing oesophageal cancer?**

□ Strongly disagree
□ Tend to disagree
□ Tend to agree
□ Strongly agree

**11. How much do you agree that frequent heartburn symptoms can increase a person’s chance of developing oesophageal cancer?**

□ Strongly disagree
□ Tend to disagree
□ Tend to agree
□ Strongly agree

**12. How much do you agree that being male can increase a person’s chance of developing oesophageal cancer?**

□ Strongly disagree
□ Tend to disagree
□ Tend to agree
□ Strongly agree

**13. How much do you agree that being female can increase a person’s chance of developing oesophageal cancer?**

□ Strongly disagree
□ Tend to disagree
□ Tend to agree
□ Strongly agree

**14. How much do you agree that being older than 50 years can increase a person’s chance of developing oesophageal cancer?**

□ Strongly disagree
□ Tend to disagree
□ Tend to agree
□ Strongly agree

**15. How much do you agree that having a close relative with oesophageal cancer can increase a person’s own chance of developing oesophageal cancer?**

□ Strongly disagree
□ Tend to disagree
□ Tend to agree
□ Strongly agree

**16. How much do you agree that drinking very hot beverages can increase a person’s chance of developing oesophageal cancer?**

□ Strongly disagree
□ Tend to disagree
□ Tend to agree
□ Strongly agree

**17. How much do you agree that chest radiotherapy (for example for breast cancer) can increase a person’s chance of developing oesophageal cancer?**

□ Strongly disagree
□ Tend to disagree
□ Tend to agree
□ Strongly agree

Beliefs about oesophageal cancer

The following statements that are sometimes made about oesophageal cancer. For each of the statements, how much do you agree or disagree?

**18. These days, many people with oesophageal cancer can expect to continue with normal activities and responsibilities.**

□ Strongly disagree
□ Tend to disagree
□ Tend to agree
□ Strongly agree

**19. Most oesophageal cancer treatment is worse than the cancer itself.**

□ Strongly disagree
□ Tend to disagree
□ Tend to agree
□ Strongly agree

**20. I would NOT want to know if I have oesophageal cancer.**

□ Strongly disagree
□ Tend to disagree
□ Tend to agree
□ Strongly agree

**21. Oesophageal cancer can often be cured.**

□ Strongly disagree
□ Tend to disagree
□ Tend to agree
□ Strongly agree

**22. Going to the doctor as quickly as possible after noticing a symptom of cancer could increase the chances of surviving.**

□ Strongly disagree
□ Tend to disagree
□ Tend to agree
□ Strongly agree

**23. Oesophageal cancer is a death sentence.**

□ Strongly disagree
□ Tend to disagree
□ Tend to agree
□ Strongly agree

**24. Out of 10 people diagnosed with oesophageal cancer, how many do you think would be**

**alive 5 years later?**

□ 0
□ 1
□ 2
□ 3
□ 4
□ 5
□ 6
□ 7
□ 8
□ 9
□ 10

Demographic background

**25. What is your gender?**

□ Male
□ Female
□ Non-binary
□ Prefer not to say

**26. What is your age?**

years

**27. What are the first 4 digits of your postal code? If you prefer not to say your postal code, you may continue with the next question.**

**28. What is your marital status?**

□ Married
□ Cohabiting
□ Divorced
□ Widowed
□ Separated
□ Single
□ Other

**29. What is the highest level of education qualification you have obtained?**

□ No education
□ Primary school
□ General secondary education (VMBO-BL)
□ Theoretical general secondary education (VMBO-T/MAVO)
□ Middle vocational education (MBO)
□ Higher secondary education (HAVO/VWO)
□ Higher vocational education (HBO)
□ Academic education (WO)

**30. What is your background?**

□ Dutch background
□ Western migration-background
□ Non-western migration-background
□ Other

**31. Have your family or close friends had oesophageal cancer?**

|  | Yes | No | Don’t know |
| --- | --- | --- | --- |
| Partner | □ | □ | □ |
| A biological family member (parent or child) | □ | □ | □ |
| Other family member | □ | □ | □ |
| Close friend | □ | □ | □ |
| Other friend | □ | □ | □ |

**32. Were you ever diagnosed with a condition of your esophagus or stomach?**

□ Yes
□ No
□ Don’t know

32.1 Shown if 32 is answered with ‘Yes’.

**32.1 Which condition was diagnosed?**
□ Barrett’s oesophagus
□ Oesophageal cancer
□ Stomach cancer
□ Oesophageal stenosis
□ Hiatus hernia
□ Other
□ Don’t remember the name
